# Integration of Ultrastructural and Computational Approaches Reveals the Protective Effect of Astaxanthin against BPA-Induced Nephrotoxicity

**DOI:** 10.3390/biomedicines11020421

**Published:** 2023-02-01

**Authors:** Refaat A. Eid, Muhammad Alaa Edeen, Mohamed A. Soltan, Mubarak Al-Shraim, Mohamed Samir A. Zaki, Saleh M. Al-Qahtani, Eman Fayad, Eman T. Salem, Waleed K. Abdulsahib, Hebatallah Emam, Hesham M. Hassan

**Affiliations:** 1 Pathology Department, College of Medicine, King Khalid University, Abha P.O. Box 62529, Saudi Arabia; 2 Cell Biology, Histology & Genetics Division, Biology Department, Faculty of Science, Zagazig University, Zagazig 44519, Egypt; 3 Department of Microbiology and Immunology, Faculty of Pharmacy, Sinai University, Ismailia 41611, Egypt; 4 Anatomy Department, College of Medicine, King Khalid University, Abha P.O. Box 62529, Saudi Arabia; 5 Department of Histology and Cell Biology, College of Medicine, Zagazig University, Zagazig 31527, Egypt; 6 Department of Child Health, College of Medicine, King Khalid University, Abha P.O. Box 62529, Saudi Arabia; 7 Department of Biotechnology, College of Sciences, Taif University, Taif 21944, Saudi Arabia; 8 Department of Basic Science, Faculty of Physical Therapy, Horus University-Egypt, New Damietta 34518, Egypt; 9 Pharmacology and Toxicology Department, College of Pharmacy, Al-Farahidi University, Baghdad 10001, Iraq; 10 Medical Biochemistry and Molecular Biology Department, Faculty of Medicine, Benha University, Benha 13518, Egypt; 11 Department of Pathology, Faculty of Medicine, Assiut University, Assiut 71515, Egypt

**Keywords:** BPA, ATX, antioxidant, kidneys, CYP2C9, dynamics

## Abstract

Background: Bisphenol A (BPA) is an environmental contaminant that can induce deleterious organ effects. Human Cytochrome P450 CYP2C9 enzyme belongs to the essential xenobiotic-metabolizing enzymes, producing ROS as a byproduct. Astaxanthin (ATX) is a powerful antioxidant that protects organs and tissues from the damaging effects of oxidative stress caused by various diseases. Aim of the study: This study investigated the possible protective impacts of ATX against BPA-induced nephrotoxicity and its underlying mechanism. Materials and methods: Kidney tissues were isolated and examined microscopically from control, protected, and unprotected groups of rats to examine the potential protective effect of ATX against nephrotoxicity. Moreover, a molecular dynamic (MD) simulation was conducted to predict the performance of ATX upon binding to the active site of P450 CYP2C9 protein receptor as a potential mechanism of ATX protective effect. Results: Implemented computational methods revealed the possible underlying mechanism of ATX protection; the protective impact of ATX is mediated by inhibiting P450 CYP2C9 through binding to its dimeric state where the RMSF value for apo-protein and ATX-complex system were 5.720.57 and 1.040.41, respectively, implicating the ATX-complex system to have lesser variance in its residues, leading to the prevention of ROS excess production, maintaining the oxidant-antioxidant balance and re-establishing the proper mitochondrial functionality. Furthermore, the experimental methods validated in silico outcomes and revealed that ATX therapy effectively restored the typical histological architecture of pathological kidney tissues. Conclusions: ATX prevents BPA-induced nephrotoxicity by controlling oxidative imbalance and reversing mitochondrial dysfunction. These outcomes shed new light on the appropriate use of ATX as a treatment or prophylactic agent for these severe conditions.

## 1. Introduction

Bisphenol A is one of the first synthetic compounds with endocrine-like capabilities and one of the most frequently used industrial molecules [1]. BPA is incorporated in manufacturing a variety of polymers, including polycarbonate as well as unsaturated polyester resin [2]. BPA is found in a wide range of industrial applications, particularly baby bottles, toys, and dental and healthcare supplies [3]. BPA has become a widespread environmental contaminant due to its high global production and use rates [4]. However, according to epidemiological studies [5], over 90% of research subjects have significant BPA levels. Based on the most recent multiple research [6,7], BPA is also abundant in food, the surroundings, and human bodily fluids. Several in vivo as well as in vitro investigations have demonstrated that BPA could deposit and impair key bodily functions [8,9,10,11]. Glucuronidation reaction, which represents one of the most fundamental detoxification mechanisms, processes the majority of BPA [12].

This research has highlighted worries about BPA’s possible involvement in the etiology of various human diseases, particularly nephrotoxicity. Mitochondria produce cellular energy and contribute to multiple metabolic pathways across the body [13]. Mitochondria, as a significant point of origin of reactive oxygen species (ROS) and reactive nitrogen species (RNS), has garnered considerable comprehensive study [14]. Consistently elevated ROS, as well as RNS levels, lead to oxidative stress and mitochondrial dysfunction [15]. Recent research has demonstrated that oxidative stress and mitochondrial dysfunction contribute to the damaging effects of BPA [16]. Conserving the integrity of mitochondrial activity and minimizing redox imbalance may be an effective treatment strategy for BPA-induced diseases. Human cytochromes P450 (P450 CYP2C9) are a class of oxygenases that catalyze the oxidative modification of both exogenous and endogenous substances [17]. These enzymes are essential for efficiently removing external chemical agents [such as poisons, medicines, and toxic substances] from the body [18]. It is known that P450 CYP2C9 contributes significantly to intracellular ROS generation and leads to oxidative damage [19]. 

Astaxanthin (ATX) is a potent antioxidant that protects organs and tissues from oxidative stress under a variety of pathological conditions [20]. Furthermore, ATX possesses significant anti-inflammatory as well as anti-apoptotic characteristics [21]. Previous findings have shown that ATX reduces mitochondrial damage induced by oxidative stress [22]. Kidney is an essential regulatory organelle that plays a critical function in helping to maintain homeostasis [23]. There is evidence that BPA concentrations in the urine of industry workers have significantly increased [24].

Furthermore, a link has been established between increased blood BPA concentrations, enhanced oxidative stress, and inflammatory markers; consequently, it is hypothesized that BPA can induce oxidative stress as well as mitochondrial malfunction, resulting in a pathological kidney damage [25].

For the first time, this research study integrated computational and ultrastructural approaches to further evaluate the potentially detrimental consequences of BPA on kidney function, and the possible defensive impact of ATX against BPA-induced nephrotoxicity.

## 2. Materials and Methods

### 2.1. Experimental Animals and Design

Thirty mature male albino rats of the Sprague–Dawley strain weighing between 150 and 200 g were housed in metal cages with sanitized bedding and free availability of food and water. Rats were kept in an environment of 24 °C and a light/dark cycle of 12 h per day. Experiments were conducted according to protocols authorized by the institutional animal ethics council (King Khalid University, Abha, Saudi Arabia). Prior to actually beginning the experiments, the experimental models were permitted one week of acclimatization.

Rats were randomly separated into three major groups (n = 10 per group). Group I: Control animals that were on a vehicle (5 ml/kg b.wt of maize oil). Experimental models were administered 50 mg/kg/bw of bisphenol A (BPA) orally in Group II (BPA group). Group III (BPA + ATX) administered ATX (25 mg/kg/b.wt) orally 1 h prior to BPA injection (50 mg/kg/b.wt). All therapies were administered once a day for five weeks.

It has been observed that the dose of BPA (50 mg/kg) stimulates oxidative stress in the kidneys of rats. The dosage and regimen used for ATX administration were determined based on prior studies demonstrating its potential to alleviate oxidative stress [26]. 

Ultimately, the kidneys were immediately detached, flash-frozen in liquid nitrogen, and preserved at −80 °C until further extensive analysis.

### 2.2. Light Microscopy 

Kidney specimens were submerged in formalin solution for 24 h before being dehydrated in ascending series of ethanol (70–100%). The samples were then xylene-cleaned and entombed in paraffin wax. Employing a microtome, specimens were sectioned at 2–3 m, and the slices were added to microscope slides. Tissue sections were then treated with Periodic acid Schiff (PAS) stain and investigated using a light microscope (Olympus, CX 43, Tokyo, Japan) [27].

### 2.3. Transmission Electron Microscopy (TEM)

According to our earlier procedures, TEM protocol was conducted [28]. Briefly, kidney specimens (1 mm^3^) from all groups were fixed in a glutaraldehyde-sodium cacodylate solution (pH = 7.4) with 2.5% glutaraldehyde. All specimens were stored at 4 °C for 4 h before being postfixed in 1% osmium tetroxide in sodium cacodylate buffer for 1 h. Samples were then dehydrated in a series of rising ethanol concentrations and washed with propylene oxide. Samples were then embedded in Spur’s resin, sectioned with an RMC Boeckeler PowerTome PC machine, as well as treated with uranyl acetate and lead citrate stains. Samples were next studied at 80 kv with a TEM (JEM, 1011, Joel Co., Tokyo, Japan) in the Electron Microscopy Unit of the Pathology Department, College of Medicine, King Khalid University.

### 2.4. In Silico Analysis of the Potential Effect of ATX on P450 CYP2C9

#### 2.4.1. System Preparation

The crystal structure of P450 CYP2C9, which was solved at a resolution of 2.55, was purchased from the protein data bank using the codes 1OG5 [29]. Employing UCSF Chimera [30], these structures were then synthesized for molecular dynamics (MD) simulations. The pH was stabilized as well as optimized at 7.5 employing PROPKA [31]. ChemBioDraw Ultra 12.1 was used to draw ATX structures [32]. As indicated in the simulation part, 20 ns MD simulations were performed on both prepared systems.

#### 2.4.2. Molecular Dynamic (MD) Simulations

Molecular dynamics (MD) simulations are increasingly being used to probe the physical motions of atoms and molecules in biological systems, offering insights that are not accessible through other methods [33]. As a result of these simulations, we can learn about the conformational changes and molecular interactions that occur throughout the dynamical evolution of biological systems [33]. All systems were simulated with MD by the GPU implementation of the PMEMD engine included in the AMBER 18 package [34].

ANTECHAMBER’s General Amber Force Field (GAFF) technique calculated the Partial atomic charge of each molecule [35]. Any system inside an orthorhombic box was implicitly solvated with TIP3P water molecules by the Leap module of the AMBER 18 package if it was located within ten of any box edge. The Leap module neutralized the systems by incorporating Na+ and Cl- counter ions. Each system was subjected to a preliminary minimization of 2000 steps using a 500 kcal/mol constraint potential, followed by a thorough minimization of 1000 steps employing the conjugate gradient approach without restraints.

During the MD simulation, all systems were heated from 0 K to 300 K over the course of 500 ps to guarantee that their atomic number and volume were identical. The solutes in the system were limited by a collision frequency of 1ps and a possible harmonic restriction of 10 kcal/mol. The systems were then heated to a constant 300 K and allowed to reach equilibrium for 500 ps. To mimic an isobaric-isothermal (NPT) ensemble, the atom count and system pressure were constant during each production simulation. Using a Berendsen barostat kept the system pressure constant at 1 bar [36].

Twenty nanoseconds were spent simulating each system using MD. The SHAKE method imposed constraints on the atoms involved in the hydrogen bonds in each simulation. The SPFP accuracy model was used in each simulation, along with a step size of 2 fs. The simulations were run in a randomly seeded isobaric-isothermal ensemble (NPT) at a constant pressure of 1 bar as well as a temperature of 300 K with a pressure-coupling constant of 2 ps and a collision frequency of 1ps using a Langevin thermostat.

#### 2.4.3. Post-MD Analysis

Trajectories obtained from MD simulations were recorded at 1 ps intervals and then analyzed with the CPPTRAJ module of the AMBER18 suite [37]. The data analysis program Origin [38] was used to produce all graphs as well as visualizations [39].

To assess ligand-binding affinities, the Poisson–Boltzmann or generalized Born and surface area continuum solvation [multipole method/surface area continuum solvation (MM/PBSA and MM/GBSA) method has been effective [40,41,42]. Using a given force field, MM/GBSA and MM/molecular PBSA’s simulations of protein–ligand complexes calculate rigorous statistical-mechanical binding free energy.

The average binding free energy was determined by 500 samples over the whole 50 ns trajectory. Different binding free energies (G) for each molecular species(complex, ligand, and receptor) can indeed be identified in the ways indicated below [43]:

(1) $$\Delta G_{bind}=G_{complex}-G_{receptor}-G_{ligand}$$

(2) $$\Delta G_{bind}=E_{gas}+G_{sol}-TS$$

(3) $$E_{gas}=E_{int}+E_{vdw}+E_{ele}$$

(4) $$G_{sol}=G_{GB}+G_{SA}$$

(5) $$G_{SA}=\gamma SASA$$

E_gas_, E_int_, E_ele_, and E_vdw_ represent the gas phase, the internal, the Coulomb, as well as the van der Waals energies, respectively. The E_gas_ was evaluated on the basis of the FF14SB force field. Solvation-free energy (G_sol_) was calculated using the energy contribution from polar and non-polar states (G_GB_ and G_GB_, respectively) (G).

A water probe with a radius of 1.4 was utilized to calculate the non-polar solvation-free energy (G_SA_) from the Solvent Accessible Surface Area (SASA) [44,45]. Conversely, the G_GB_ contribution was determined by solving the GB equation. Items S and T represent the solute’s total entropy as well as temperature, correspondingly.

### 2.5. Statistical Analysis

The data are displayed as means ± SEM. Statistical significance was determined by employing SPSS (SPSS v. 20.0, SPSS Inc., Chicago, IL, USA) software and independent-sample *t*-tests. When *p* < 0.05, data were deemed statistically significant. Figures indicate the number of duplicates utilized for the analysis.

## 3. Results

### 3.1. Light Microscope

**Figure 1:**
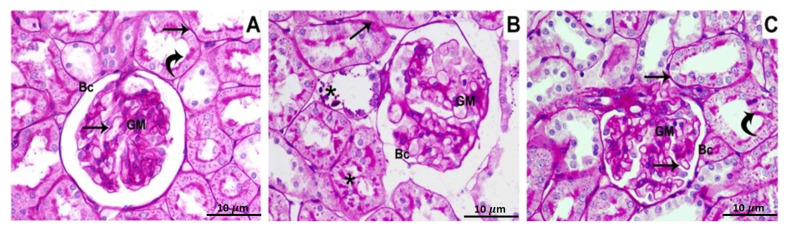
Photomicrographs of the renal cortex stained with PAS show, Control group (**A**): The glomerular and tubular basement membranes (arrows), glomerular matrix (GM), and the brush border of the proximal convoluted tubules (curved arrows), all showed a strong PAS-positive reaction; (**B**) BPA-treated group: the tubular basement membrane (arrow) thickening, elevated PAS positivity in the mesangial matrix (GM), and damage of the renal tubule brush border (stars); (**C**) BPA and ATX-treated groups have thin glomerular and tubular basement membranes (arrows), with normal mesangial matrix deposition (GM). The brush border of the renal tubules has a PAS-positive reaction (curved arrows). Bowman’s capsule was pointed out by BC. 10 µm scale bar.

PAS-stained specimens of the control group’s renal cortex reveal that the glomerular capillaries, as well as renal tubules, have normal, thin basement membranes and also that the mesangial matrix deposits are perfectly normal (Figure 1A). Sections from BPA group show a thickening of the glomerular basement membrane, mesangial expansion, cellular proliferation, and a discontinuous brush border of the renal tubules (Figure 1B). Tissue specimen of group 3, which was administered BPA plus ATX, exhibit thin glomerular and tubular membranes, as well as a characteristic mesangial matrix and brush borders of the tubules (Figure 1C).

### 3.2. Transmission Electron Microscope (TEM)

**Figure 2:**
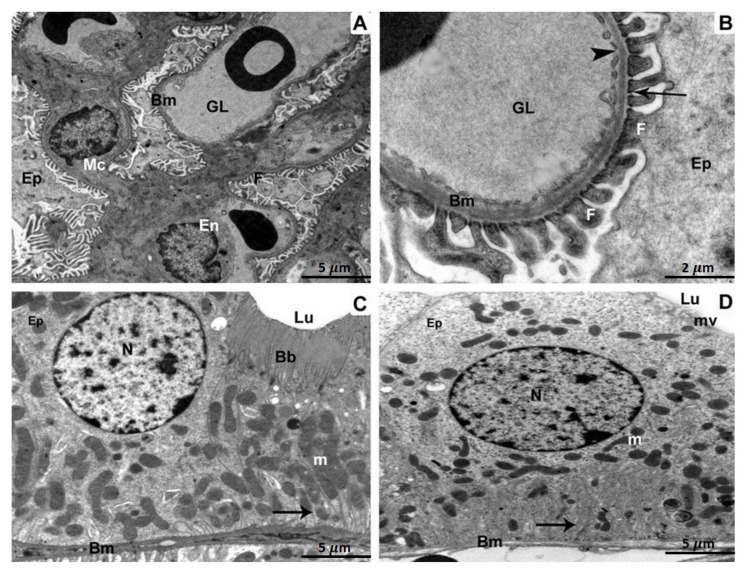
Kidney specimens of the control group are depicted in TEM as follows: (**A**) A glomerulus with glomerular capillaries (GL), basement membranes (Bm), endothelium (En), mesangial cells (Mc), podocytes (Ep), as well as foot processes (F). 5 µm scale bar; (**B**) A more in-depth view of the glomerular basement membrane (Bm) revealing its three distinct layers: lamina rara interna, rara externa, and densa. Foot processes (F), fenestration (arrow heads), thin diaphragms (arrows), as well as podocytes (Ep) are all illustrated (Ep). At 2 µm scale bar, section (**C**) of a proximal convoluted tubule reveals cuboidal epithelial cells (Ep) resting on the basement membrane (Bm) and the tubular lumen brush border (Lu) (Bb). The nucleus (N), mitochondria (m), in addition to infolding membranes are all distinguished characteristics (arrow). 5 µm scale bar; (**D**) A section through a distal convoluted tubule revealing cuboidal epithelial cells (Ep) resting on a basement membrane and displaying short microvilli (mv) (Bm). The infolding membranes, the nucleus, and the mitochondria are all prominent characteristics (arrow). 5 µm scale bar.

Kidney specimens of the control, as well as ATX-treated rat groups, exhibited a regular glomerulus with its basement membranes, capillary endothelium, and mesangial cells, in addition to podocytes with their foot processes (Figure 2A). Relatively high magnification showed the three glomerular basement membrane layers: an inner layer (lamina rara interna), an exterior layer (lamina rara externa), as well as a middle layer (lamina densa) containing thin diaphragms (Figure 2B). In a proximal-convoluted tubule cross-section, the epithelium tissue that lines the tube and resting on the basement membrane were observable. Inside the tubule, there were also brush-border microvilli, regular mitochondria, a perfectly intact nucleus, as well as basal infolding membranes (Figure 2C). Epithelium with small microvilli, undamaged mitochondria, a typical nucleus, in addition to infolding membranes were seen in a distal-convoluted tubule cross-section (Figure 2D). 

**Figure 3:**
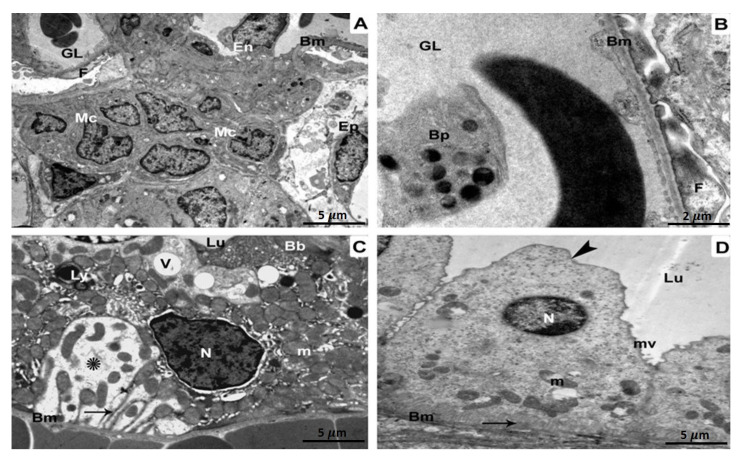
TEM investigation of kidney specimens of BPA-treated group. (**A**) The basement membrane (Bm) as well as glomerular capillaries of a pleomorphic glomerulus, are wrinkled and branching (GL). Podocytes, in addition to mesangial cells (MCs) proliferate, and the endothelium (En) is injured5 µm scale bar; (**B**) A more powerful magnification is displaying glomerular basement membranes (Bm) with fusion foot processes (F) and some blood platelets (Bp) within the glomerular lumen (GL) 2 µm scale bar; (**C**) Abnormal aggregation of lysosomes (Ly) as well as vacuoles (V) in the Epithelium of a distorted proximal tubule (Ep). Brush borders (Bb), pyknotic nuclei (N), enlarged mitochondria (m), lipid droplets (L), and also the tubular basement membrane (BM) are all disrupted (Bm). There is evidence of both damaged cytoplasm (shown by the star) and infolding membranes (represented by the arrow). Magnification: 5 µm scale bar (**D**) A districted epithelium (Ep) organelles lying on the basement membrane in the lumen (Lu) of a disrupted distal tubule with an enlarged apical region of Epithelium (arrowhead) (Bm). Vacuoles (V), dysfunctional mitochondria (m), as well as deteriorated nuclei (N) are also observable. At 5 µm scale bar, we can clearly detect disrupted cytoplasm (star) as well as infolding membranes (arrow).

The glomerulus in the BPA-treated group underwent morphological alterations that included aberrant glomeruli with wrinkling and branching basement membranes. Additionally observed were damaged capillary endothelium, mesangial expansion, and cellular proliferation (Figure 3A). The glomerulus was magnified at a higher magnification, and the basement membranes were shown to have wrinkled. The foot processes also appeared to have fused (Figure 3B). A deformed proximal tubule showed several lysosomes and vacuoles in its epithelial cells. In addition, there were lipid droplets, enlarged mitochondria, damaged brush borders, and irregular pyknotic nuclei (Figure 3C). An aberrant distal tubule displayed disorganized organelles and an abnormal epithelial lining lying on the basement membrane. Multiple vacuoles, fragmented mitochondria, and atrophied nuclei were also seen (Figure 3D). 

**Figure 4:**
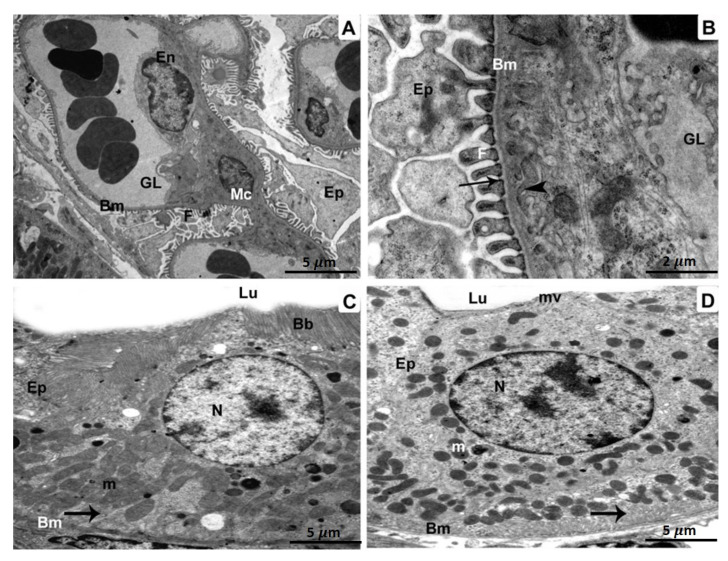
Specimens of BPA- and ATX-treated group, as seen in TEM: A glomerulus containing glomerular capillaries (GL), a basement membrane (Bm), endothelium (En), a mesangial cell (MC), and podocytes (Ep) with foot processes. (**A**). 5 µm scale bar; (**B**) A greater magnification showing a glomerular capillary (GL) bordered by a basement membrane (Bm) 2 µm scale bar. A three-layered glomerular membrane with fenestrations (arrowheads), foot processes (F), as well as podocytes (Ep) is shown. Epithelial cells (Ep) are seen in a cross-section of a proximal tubule (**C**) resting on the basement membrane (Bm). The nucleus (N), in addition to infolding membranes (arrow), are both present, as are the mitochondria (M) (m). 5 µm scale bar; Epithelial cells (Ep) resting on the basement membrane, as seen in a cross-section of a distal tubule (**D**) (Bm). We can observe the undamaged, intact nucleus (N), infolding membranes (arrow), as well as functioning intact mitochondria (m). 5 µm scale bar.

A glomerulus from the BPA plus ATX-treated group displaying the capillary endothelium, mesangial cells, and podocytes with foot processes (Figure 4A). Glomerulus with higher magnification revealed minimally thickened capillary endothelium. There were visible foot processes, fenestrations, the three layers of the glomerular basement membrane, thin diaphragms, and podocytes (Figure 4B). A proximal tubule exhibited intact mitochondria, a healthy nucleus, and basal infoldings in addition to the epithelial cells that line the tubule (Figure 4C). Epithelial cells sitting on an enfolded basement membrane were visible in a distal tubule, together with intact mitochondria and a proper nucleus (Figure 4D). 

### 3.3. In Silico Drug Discovery

#### 3.3.1. Molecular Dynamic and System Stability 

**Figure 5:**
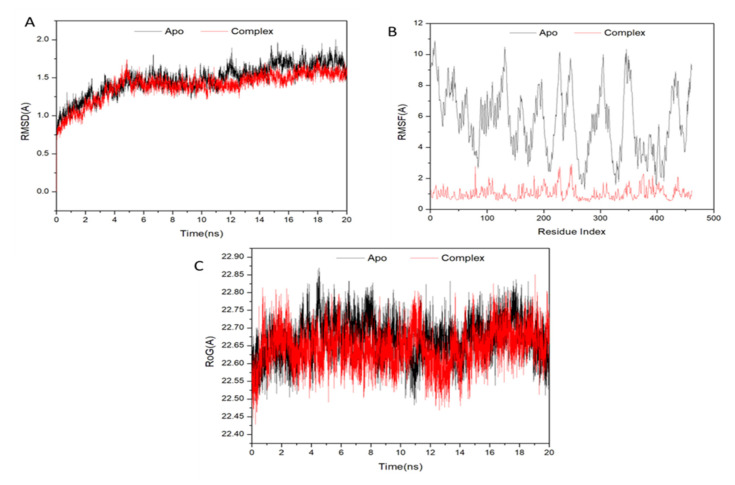
(**A**) RMSD of Cα atoms of the protein backbone atoms; (**B**) RMSF of each residue of the protein backbone Cα atoms; (**C**) Radius of Gyration (ROG) of Cα atoms of protein residues of the backbone atoms relative (black) to the starting minimized over 20 ns for the P450 CYP2C9 protein with ligand ATX (red).

A molecular dynamic simulation was performed to predict how effectively the extracted compounds would function after binding to a protein’s active site, considering their interaction and stability [46]. It is essential to verify the stability of the system in order to track the source of any disruptions and avoid any artifacts that may have resulted from the simulation. In order to assess the stability of systems throughout the course of 20 ns simulations, this study used root–mean–square deviation (RMSD). The average root–mean–square deviation (RMSD) for all frames in the apo-protein system, as well as the ATX-complex system, was 1.47 0.20 and 1.39 0.20, respectively (Figure 5A). These results show that ATX-protein complex system achieved a significantly more stable conformation compared to the other investigated system.

In order to analyze residue behavior and their correlation to the ligand during MD simulation, it is crucial to evaluate structural flexibility in the protein after ligand binding [47]. The root–mean–square fluctuation (RMSF) method was used to study the impact of inhibitor binding to important targets during 20 ns simulations on protein residue fluctuations. The average RMSFs for apo-protein and also ATX-complex system were 5720.57 and 1040.41, respectively. Figure 5B shows the cumulative alterations in residues across all systems. ATX-protein complex system was shown to have lesser variance in its residues based on these values than the other system inhibitions.

Radius of gyration (Rg) indicates the compactness and simulated stability of protein structures. Figure 5C demonstrates that Rg values of apo-protein and complex containing ATX were 22,660.05 and 22,640.05, respectively. Rg of ligand-bonded protein had a more rigid structure compared to Apo-protein.

#### 3.3.2. Binding Interaction Mechanism Based on Binding Free Energy Calculation

Small-molecule free binding energies to biological macromolecules are often estimated using the molecular mechanics’ energy methodology (MM/GBSA), which includes the generalized Born and surface area continuum solvation, is a common approach that may be more accurate compared to docking scores [48]. The binding free energies were calculated using snapshots of the systems’ trajectories extracted using the MM-GBSA tool in AMBER18. Table 1 shows that, with the exception of Gsolv, each recorded computed energy components have significantly negative values, indicative of positive interactions. ATX had a binding affinity of −79.63 kcal/mol for proteins, as measured by the data.

Examining the various energy contributions that led to the reported binding free energies shows that the interactions between ATX and the P450 CYP2C9 protein receptor residues are regulated by the higher positive Vander Waals energy components (Table 1).

#### 3.3.3. Determination of the Essential Amino Acid Residues Implicated in Ligand Binding and Complex Formation

**Figure 6:**
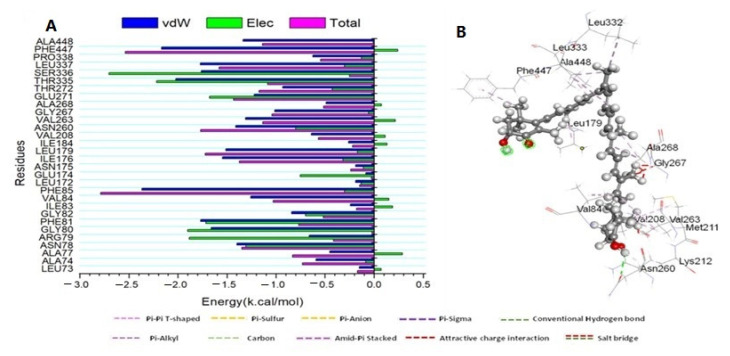
(**A**) Per-residue decomposition plots showing the energy contributions to the binding and stabilization of the Astaxanthin at the catalytic active site of human cytochrome P450 CYP2C9 protein receptor; (**B**) Corresponding inter-molecular interaction of the Astaxanthin to the catalytic active site of human cytochrome P450 CYP2C9 protein receptor.

To establish a comprehensive residue understanding involved in human cytochrome P450 CYP2C9 protein receptor inhibition, the amount of energy involved when ATX binds to these enzymes has been decomposed into the participation of specific site residues. Figure 6 illustrates that the significant favorable contribution of ATX compound to the P450 CYP2C9 protein receptor is predominantly detected from residues Leu 73 (−0.148 kcal/mol), Ala 74 (−0.59 kcal/mol), Ala 77 (−0.449 kcal/mol), Asn 78 (−1.39 kcal/mol), Arg 79 (−0.662 kcal/mol), Gly 80 (−1.662 kcal/mol), Phe 81 (−1.768 kcal/mol), Gly82 (−0.839 kcal/mol), Ile83 (−0.239 kcal/mol), Val 84 (−1.257 kcal/mol), Phe 85 (−2.369 kcal/mol), Leu 172 (−0.187 kcal/mol), Ile 176 (−1.546 kcal/mol), Leu 179 (−1.51 kcal/mol), Ile 184 (−0.258 kcal/mol), Val 208 (−0.638 kcal/mol), Asn 260 (−1.411 kcal/mol), Val 263 (−1.31 kcal/mol), Gly 267 (−1.011 kcal/mol), Ala 268 (−0.489 kcal/mol), Glu 271 (−1.223 kcal/mol), Thr 272 (−0.931 kcal/mol), Thr 335 (−2.023 kcal/mol), Ser 336 (−1.761 kcal/mol), Leu 337 (−1.772 kcal/mol), Pro 338 (−0.622 kcal/mol), Phe447 (−2.168 kcal/mol), and Ala448 (−1.335 kcal/mol).

## 4. Discussion

Kidneys are vital organs in the human body, responsible for excreting metabolites and toxic chemicals, maintaining water balance and electrolyte balance, as well as acid-base balance, regulating blood pressure, promoting erythropoiesis, and activating vitamin D [49]. Renal activities support the homeostasis of the internal environment and permit regular metabolic activity [50]. As a documented environmental contaminant, BPA poses a substantial hazard to public health [5]. BPA has been found in food samples, environmental samples, human blood, urine, and breast milk [51]. Because specific tissues and organs are vulnerable to the BPA harmful impacts, the existence of BPA in biological specimens is a considerable threat [52]. ATX is an antioxidant that has gained widespread interest for its potential to cure oxidative and inflammatory diseases [53].

ATX has shown superiority in the protective effects against several kidney disorders, including nephrolithiasis, renal fibrosis, and acute kidney injury (AKI) [54,55]. On the other hand, BPA can exert nephrotoxicity by inducing oxidative stress, where it was reported that BPA aggravates renal apoptosis and necroptosis via oxidative stress and PI3K/AKT pathway [56]. The modulating effect of ATX on BPA cytotoxicity has been reported in several studies on multiple cell levels where ATX was reported to enhance follicles development through controlling oxidative stress induced by BPA in cultured follicles [57]. Moreover, autophagic cell death, as a result of BSA, was significantly restored upon a treatment with ATX [58]. In addition to that, ATX showed protective roles against nephrotoxicity in rats with induced renovascular occlusion where caspase-3 and oxidative stress markers where significantly decreased in ATX protected group [55]. Here, we aimed to confirm the ATX protection against BPA-induced nephrotoxicity where we employed a comparative analysis on the output of light and transmission electron microscopes to achieve that purpose. Moreover, a computational analysis was performed to investigate a potential mechanism for this protective effect. The in silico study recommended the interaction between ATX and P450 CYP2C9 as a potential mechanism for the ATX protective effect where future biochemical assessment is required to confirm that mechanism.

In order to comprehend the detrimental impacts of BPA and ATX protective effects on the kidneys, we investigated the consequences of BPA and ATX exposure on the renal impairment and protection of animal models. This research also demonstrated the underlying potential mechanism of ATX to influence renal functions, which was uncovered using novel computational approaches. Our findings demonstrated that frequent BPA exposure for five weeks led to detrimental effects on kidneys; these effects were initially validated by interpreting the findings of a light microscope which revealed the glomerular basement membrane thickening, mesangial expansion, cellular proliferation, as well as a discontinuity of the brush border of the renal tubules in the tissue specimens of animal models which were treated with BPA only. On the contrary, the potential protective effect of ATX was validated by interpreting the findings of ATX on kidney tissues of animal models which were on ATX+BPA, as these findings demonstrated a thin glomerular and tubular membranes, along with a typical mesangial matrix and brush border of the tubules, which confirm the restoration of normal tissue architecture. Furthermore, the histopathological examination using TEM further confirmed the above-mentioned effects of BPA and ATX by revealing that the glomerulus in the BPA-treated group underwent morphological alterations that included aberrant glomeruli with wrinkling and branching basement membranes. Additionally observed were damaged capillary endothelium, mesangial expansion, and cellular proliferation. In contrast, investigating tissue specimens of BPA + ATX-treated group verified the protective effects of ATX, by displaying the capillary endothelium, mesangial cells, and podocytes with foot processes; a proximal tubule exhibited intact mitochondria, a healthy nucleus, and basal infoldings in addition to the epithelial cells that line the tubule; epithelial cells sitting on an enfolded basement membrane were visible in a distal tubule, together with intact mitochondria and a proper nucleus

To our knowledge, this study is the first to investigate the potential detrimental and protective effects of BPA and ATX, respectively, in the context of ultrastructural pathology and computational approaches. Interestingly, our findings are in agreement with several previously conducted studies which focused on the same objective but mainly in the context of biochemical analysis. For instance, WeiJiang et al. [26] found that ATX protects against BPA-induced kidney injury, in particular, by controlling oxidative imbalance and enhancing mitochondrial function. Moreover, Li L et al. [59] reported that ATX is an appropriate and efficient protective agent in renal injury produced by OTA exposure; therefore suggested that his research findings offer theoretical justification for ATX’s participation in other mycotoxin-induced damages. Moreover, in vitro research studies have demonstrated that BPA induces mitochondrial damage in renal proximal tubular epithelial cells [60]. ATX therapy ameliorated these adverse effects of BPA [61]. Oxidative stress refers to an imbalance between ROS production and antioxidant defenses that results in oxidative stress [62]. 

Once a body is under oxidative stress conditions, ROS as well as RNS levels rise, surpassing the body’s capacity to adequately scavenge free radicals, resulting in DNA oxidative damage and aberrant protein expression and eventually causing physical damage [63]. Earlier studies established that oxidative stress is one of the primary mechanisms by which BPA impacts human cells [64]. Mitochondria are semiautonomous organelles in various mammalian cell types; they contain their own DNA and genetic systems [65]. These findings revealed that renal mitochondrial dysfunction generated by BPA was a crucial mechanism for nephrotoxicity. A small count of electrons prematurely deoxygenate oxygen to produce ROS and RNS during mitochondrial oxidative phosphorylation, resulting in oxidative stress [66]. Mitochondria have a significant impact as a crucial component of the innate immune pathway, as well as studies have revealed that compromised mitochondrial integrity induces inflammatory responses and diseases [55]. Mitochondria are implicated in apoptosis and provide energy to cells, and the mitochondrial apoptosis pathway is among the conventional apoptotic mechanisms [56]. According to this present research, BPA-induced oxidative stress, as well as cell death, were all intrinsically connected to mitochondrial dysfunction. This study indicated that ATX was co-administered with BPA-exposed rats in order to alleviate the nephrotoxicity triggered by BP.

P450 CYP2C9 belongs to a family of heme-thiolate enzymes implicated in the oxidative metabolism of numerous endogenous as well as exogenous lipophilic compounds [67]. Poor coupling of the P450 CYP2C9 catalytic cycle causes the continual creation of ROS, which interferes with signaling pathways and other cellular activities [68]. The production of ROS by P450 CYP2C9 is closely regulated by the regulation of gene transcription and, indeed, the modification of connections between protein parts of the monooxygenase, which influences its function, coupling, as well as stability [67]. A failure of these systems may lead to an influx of ROS, which may participate in lipid peroxidation and oxidative stress [66]. Consequently, in silico analysis was used to discover the potential underlying mechanisms of ATX protection against BPA-induced nephrotoxicity. Regarding molecular dynamic and system stability, ATX- P450 CYP2C9 complex system obtained a significantly more stable conformation than the other examined systems. Moreover, our findings demonstrated that the ATX- P450 CYP2C9 complex system has a low residue variation compared to other inhibitions. Additionally, Rg of ligand-bonded protein was shown to have a more rigid structure than Apo-protein. 

Our findings revealed that ATX-P450 CYP2C9 complex system has the most significant performance, interaction, and stability upon binding to the active site of P450 CYP2C9. Moreover, ATX-P450 CYP2C9 complex showed the most significant protein structural flexibility following ligand binding, in addition to the most significant compactness and simulated stability. Furthermore, the findings of MM-GBSA tool in AMBER18 reported that the computed energy components (except for Gsolv) had significant negative values, demonstrating positive interactions, and ATX binding affinity to protein was −79.63 kcal/mol. Additionally, we found that interactions between ATX and P450 CYP2C9 protein receptor residues are mediated by the greater positive van der Waals energy components, as demonstrated by a comprehensive analysis of each individual energy contribution that resulted in the documented binding free energies. These results indicated a stable binding interaction between ATX and P450 CYP2C9.

Ultimately, the in silico methods verified the potential protective effect of ATX and demonstrated its underlying mechanism. Herein, we recommend that ATX-protective role against BPA-induced nephrotoxicity is regulated by coupling or binding to P450 CYP2C9, which consequently leads to restoring and maintaining oxidate balance and prevents excessive ROS generation and oxidative stress, which consider the main reason for mitochondrial dysfunction, kidneys tissue damage and nephrotoxicity. Collectively, the findings of our research study consider additional significant evidence of the efficient protective role of ATX against BPA-induced nephrotoxicity, as they agree with the previous results of several studies which aimed to investigate the same function. However, from other perspectives, mainly biochemistry, our study is the first to validate these earlier findings in the context of ultrastructural pathology and novel computational approaches.

## 5. Conclusions

BPA exposure is detrimental to the kidneys. Mitochondrial dysfunction is a significant mediator of BPA-induced nephrotoxicity. This study revealed that ATX inhibits BPA-induced nephrotoxicity, and a comparative analysis of the output from light and transmission electron microscope confirmed that finding. Moreover, a computational analysis recommended the interaction between astaxanthin and P450 CYP2C9 as a potential mechanism for the ATX protective effect where future biochemical assessment is required to confirm that mechanism.

## Figures and Tables

**Table 1:** Calculated energy binding for the ATX compounds against P450 CYP2C9 protein receptor.

Energy Components (kcal/mol)					
Complex	ΔE_vdW_	ΔE_elec_	ΔG_gas_	ΔG_solv_	ΔG_bind_
**ATX**	−81.27 ± 0.30	−42.58 ± 0.43	−123.85 ± 0.51	44.21 ± 0.27	−79.63 ± 0.44

∆E_vdW_ = van der Waals energy; ∆E_elec_ = electrostatic energy; ΔG_gas_ = gas phase interaction; ∆G_solv_ = solvation free energy; ∆G_bind_ = calculated total binding free energy.

## Data Availability

Data are available with the corresponding authors upon reasonable request.

## References

[1] Staniszewska M., Graca B., Nehring I. (2016). The fate of bisphenol A, 4-tert-octylphenol and 4-nonylphenol leached from plastic debris into marine water—Experimental studies on biodegradation and sorption on suspended particulate matter and nano-TiO_2_. Chemosphere.

[2] Liguori F., Moreno-Marrodan C., Barbaro P. (2020). Biomass-derived chemical substitutes for bisphenol A: Recent advancements in catalytic synthesis. Chem. Soc. Rev..

[3] Ejaredar M., Lee Y., Roberts D.J., Sauve R., Dewey D. (2017). Bisphenol A exposure and children’s behavior: A systematic review. J. Expo. Sci. Environ. Epidemiol..

[4] lo Turco V., Potortì A.G., ben Mansour H., Dugo G., di Bella G. (2020). Plasticizers and BPA in spices and aromatic herbs of Mediterranean areas. Nat. Prod. Res..

[5] Calafat A.M., Ye X., Wong L.Y., Reidy J.A., Needham L.L. (2008). Exposure of the U.S. population to Bisphenol A and 4-tertiary-octylphenol: 2003–2004. Environ. Health Perspect..

[6] Mouneimne Y., Nasrallah M., Khoueiry-Zgheib N., Nasreddine L., Nakhoul N., Ismail H., Abiad M., Koleilat L., Tamim H. (2017). Bisphenol A urinary level, its correlates, and association with cardiometabolic risks in Lebanese urban adults. Environ. Monit. Assess..

[7] Fenichel P., Dechaux H., Harthe C., Gal J., Ferrari P., Pacini P., Wagner-Mahler K., Pugeat M., Brucker-Davis F. (2012). Unconjugated bisphenol A cord blood levels in boys with descended or undescended testes. Hum. Reprod..

[8] Kabuto H., Hasuike S., Minagawa N., Shishibori T. (2003). Effects of bisphenol A on the metabolisms of active oxygen species in mouse tissues. Environ. Res..

[9] Moghaddam H.S., Samarghandian S., Farkhondeh T. (2015). Effect of bisphenol A on blood glucose, lipid profile and oxidative stress indices in adult male mice. Toxicol. Mech. Methods.

[10] Tiwari D., Kamble J., Chilgunde S., Patil P., Maru G., Kawle D., Bhartiya U., Joseph L., Vanage G. (2012). Clastogenic and mutagenic effects of bisphenol A: An endocrine disruptor. Mutat. Res./Genet. Toxicol. Environ. Mutagen..

[11] Xia W., Jiang Y., Li Y., Wan Y., Liu J., Ma Y., Mao Z., Chang H., Li G., Xu B. (2014). Early-life exposure to bisphenol a induces liver injury in rats involvement of mitochondria-mediated apoptosis. PLoS ONE.

[12] Knizatova N., Tokárová K., Greifová H., Jambor T., Massányi P., Lukáč N. (2020). Bisphenol A Analogues: A Brief Review of their Occurrence in Food, Biological Samples and Endocrine Effects. Arch. Ecotoxicol..

[13] Tao S., Luo Y., He B., Liu J., Qian X., Ni Y., Zhao R. (2016). Paraoxonase 2 modulates a proapoptotic function in LS174T cells in response to quorum sensing molecule N-[3-oxododecanoyl]-L-homoserine lactone. Sci. Rep..

[14] Li J., Li N., Yan S., Lu Y., Miao X., Gu Z., Shao Y. (2019). Melatonin attenuates renal fibrosis in diabetic mice by activating the AMPK/PGC1α signaling pathway and rescuing mitochondrial function. Mol. Med. Rep..

[15] Islam M.T. (2017). Oxidative stress and mitochondrial dysfunction-linked neurodegenerative disorders. Neurol. Res..

[16] Uzunhisarcikli M., Aslanturk A. (2019). Hepatoprotective effects of curcumin and taurine against bisphenol A-induced liver injury in rats. Environ. Sci. Pollut. Res..

[17] Zangar R.C., Davydov D.R., Verma S. (2004). Mechanisms that regulate production of reactive oxygen species by cytochrome P450. Toxicol. Appl. Pharmacol..

[18] Gonzalez F.J. (2005). Role of cytochromes P450 in chemical toxicity and oxidative stress: Studies with CYP2E1. Mutat. Res. Mol. Mech. Mutagen..

[19] Acharya P., Liao M., Engel J.C., Correia M.A. (2011). Liver Cytochrome P450 3A endoplasmic reticulum-associated degradation: A major role for the P97 AAA ATPase in cytochrome P450 3A extraction into the cytosol. J. Biol. Chem..

[20] Li J., Wang F., Xia Y., Dai W., Chen K., Li S., Liu T., Zheng Y., Wang J., Lu W. (2015). Astaxanthin Pretreatment Attenuates Hepatic Ischemia Reperfusion-Induced Apoptosis and Autophagy via the ROS/MAPK Pathway in Mice. Mar. Drugs.

[21] Kohandel Z., Farkhondeh T., Aschner M., Pourbagher-Shahri A.M., Samarghandian S. (2022). Anti-inflammatory action of astaxanthin and its use in the treatment of various diseases. Biomed. Pharmacother..

[22] Yu T., Dohl J., Chen Y., Gasier H.G., Deuster P.A. (2019). Astaxanthin but not quercetin preserves mitochondrial integrity and function, ameliorates oxidative stress, and reduces heat-induced skeletal muscle injury. J. Cell. Physiol..

[23] van Westing A.C., Küpers L.K., Geleijnse J.M. (2020). Diet and Kidney Function: A Literature Review. Curr. Hypertens. Rep..

[24] Hines C.J., Christianson A.L., Jackson M.V., Ye X., Pretty J.R., Arnold J.E., Calafat A.M. (2018). An Evaluation of the Relationship among Urine, Air, and Hand Measures of Exposure to Bisphenol A [BPA] in US Manufacturing Workers. Ann. Work. Expo. Health.

[25] Quiroga B. (2021). Strategies to Protect Dialysis Patients against Bisphenol A. Biomolecules.

[26] Jiang W., Zhao H., Zhang L., Wu B., Zha Z. (2020). Maintenance of mitochondrial function by astaxanthin protects against bisphenol A-induced kidney toxicity in rats. Biomed. Pharmacother..

[27] Janardhan K.S., Jensen H., Clayton N.P., Herbert R.A. (2018). Immunohistochemistry in Investigative and Toxicologic Pathology. Toxicol. Pathol..

[28] Eid R.A., Bin-Meferij M.M., El-Kott A.F., Eleawa S.M., Zaki M.S.A., Al-Shraim M., El-Sayed F., Eldeen M.A., Alkhateeb M.A., Alharbi S.A. (2020). Exendin-4 Protects Against Myocardial Ischemia-Reperfusion Injury by Upregulation of SIRT1 and SIRT3 and Activation of AMPK. J. Cardiovasc. Transl. Res..

[29] Katoh H., Negishi M. (2003). RhoG activates Rac1 by direct interaction with the Dock180-binding protein Elmo. Nature.

[30] Pettersen E.F., Goddard T.D., Huang C.C., Couch G.S., Greenblatt D.M., Meng E.C., Ferrin T.E. (2004). UCSF Chimera—A visualization system for exploratory research and analysis. J. Comput. Chem..

[31] Li H., Robertson A.D., Jensen J.H. (2005). Very fast empirical prediction and rationalization of protein pK_a_ values. Proteins Struct. Funct. Genet..

[32] Evans D.A. (2014). History of the Harvard ChemDraw Project. Angew. Chem.-Int. Ed..

[33] Hospital A., Goñi J.R., Orozco M., Gelpí J.L. (2015). Molecular dynamics simulations: Advances and applications. Adv. Appl. Bioinform. Chem..

[34] Lee T.-S., Cerutti D.S., Mermelstein D., Lin C., LeGrand S., Giese T.J., Roitberg A., Case D.A., Walker R.C., York D.M. (2018). GPU-Accelerated Molecular Dynamics and Free Energy Methods in Amber18: Performance Enhancements and New Features. J. Chem. Inf. Model..

[35] Wang J., Wang W., Kollman P.A., Case D.A. (2006). Automatic atom type and bond type perception in molecular mechanical calculations. J. Mol. Graph. Model..

[36] Berendsen H.J.C., Postma J.P.M., van Gunsteren W.F., Dinola A., Haak J.R. (1984). Molecular dynamics with coupling to an external bath. J. Chem. Phys..

[37] Roe D.R., Cheatham T.E. (2013). PTRAJ and CPPTRAJ: Software for Processing and Analysis of Molecular Dynamics Trajectory Data. J. Chem. Theory Comput..

[38] Seifert E. (2014). OriginPro 9.1: Scientific Data Analysis and Graphing Software—Software Review. J. Chem. Inf. Model..

[39] Meng E.C., Pettersen E.F., Couch G.S., Huang C.C., Ferrin T.E. (2006). Tools for integrated sequence-structure analysis with UCSF Chimera. BMC Bioinform..

[40] Hayes J.M., Archontis G. (2012). MM-GB[PB]SA Calculations of Protein-Ligand Binding Free Energies. Molecular Dynamics—Studies of Synthetic and Biological Macromolecules.

[41] Ylilauri M., Pentikäinen O.T. (2013). MMGBSA As a Tool To Understand the Binding Affinities of Filamin–Peptide Interactions. J. Chem. Inf. Model..

[42] Kollman P.A., Massova I., Reyes C., Kuhn B., Huo S., Chong L., Lee M., Lee T., Duan Y., Wang W. (2000). Calculating Structures and Free Energies of Complex Molecules: Combining Molecular Mechanics and Continuum Models. Accounts Chem. Res..

[43] Hou T., Wang J., Li Y., Wang W. (2011). Assessing the Performance of the MM/PBSA and MM/GBSA Methods. 1. The Accuracy of Binding Free Energy Calculations Based on Molecular Dynamics Simulations. J. Chem. Inf. Model..

[44] Sitkoff D., Sharp K.A., Honig B. (1994). Accurate Calculation of Hydration Free Energies Using Macroscopic Solvent Models. J. Phys. Chem..

[45] Greenidge P.A., Kramer C., Mozziconacci J.-C., Wolf R.M. (2013). MM/GBSA Binding Energy Prediction on the PDBbind Data Set: Successes, Failures, and Directions for Further Improvement. J. Chem. Inf. Model..

[46] Mirzaei S., Eisvand F., Hadizadeh F., Mosaffa F., Ghasemi A., Ghodsi R. (2020). Design, synthesis and biological evaluation of novel 5,6,7-trimethoxy-N-aryl-2-styrylquinolin-4-amines as potential anticancer agents and tubulin polymerization inhibitors. Bioorganic Chem..

[47] Machaba K.E., Mhlongo N.N., Soliman M.E.S. (2018). Induced Mutation Proves a Potential Target for TB Therapy: A Molecular Dynamics Study on LprG. Cell Biochem. Biophys..

[48] Cournia Z., Allen B., Sherman W. (2017). Relative Binding Free Energy Calculations in Drug Discovery: Recent Advances and Practical Considerations. J. Chem. Inf. Model..

[49] Franzin R., Stasi A., Fiorentino M., Stallone G., Cantaluppi V., Gesualdo L., Castellano G. (2020). Inflammaging and Complement System: A Link Between Acute Kidney Injury and Chronic Graft Damage. Front. Immunol..

[50] Mitrakou A. (2011). Kidney: Its impact on glucose homeostasis and hormonal regulation. Diabetes Res. Clin. Pract..

[51] Anet A., Olakkaran S., Purayil A.K., Puttaswamygowda G.H. (2019). Bisphenol A induced oxidative stress mediated genotoxicity in Drosophila melanogaster. J. Hazard. Mater..

[52] Beltifa A., Feriani A., Macherki M., Ghorbel A., Ghazouani L., Di Bella G., Sire O., Van Loco J., Reyns T., Ben Mansour H. (2018). Persistent plasticizers and bisphenol in the cheese of Tunisian markets induced biochemical and histopathological alterations in male BALB/c mice. Environ. Sci. Pollut. Res..

[53] Mularczyk M., Michalak I., Marycz K. (2020). Astaxanthin and other Nutrients from *Haematococcus pluvialis*—Multifunctional Applications. Mar. Drugs.

[54] Alugoju P., Swamy V.K.D.K., Anthikapalli N.V.A., Tencomnao T. (2022). Health benefits of astaxanthin against age-related diseases of multiple organs: A comprehensive review. Crit. Rev. Food Sci. Nutr..

[55] Arslan E., Turk H., Caglayan M., Turkmenoglu T.T., Gonel A., Tayman C. (2021). Protective Effects of Astaxanthin on Nephrotoxicity in Rats with Induced Renovascular Occlusion. Comb. Chem. High Throughput Screen..

[56] Chen H., Zhang Y., Zou M., Qi X., Xu S. (2022). Bisphenol A aggravates renal apoptosis and necroptosis in selenium-deficient chickens via oxidative stress and PI3K/AKT pathway. J. Cell Physiol..

[57] Li Y., Dong Z., Liu S., Gao F., Zhang J., Peng Z., Wang L., Pan X. (2022). Astaxanthin improves the development of the follicles and oocytes through alleviating oxidative stress induced by BPA in cultured follicles. Sci. Rep..

[58] Lim S.R., Kim D.W., Sung J., Kim T.H., Choi C.H., Lee S.J. (2021). Astaxanthin inhibits autophagic cell death induced by bisphenol a in human dermal fibroblasts. Antioxidants.

[59] Li L., Chen Y., Jiao D., Yang S., Li L., Li P. (2020). Protective effect of astaxanthin on ochratoxin A-induced kidney injury to mice by regulating oxidative stress-related Nrf2/Keap1 pathway. Molecules.

[60] Barbonetti A., Castellini C., di Giammarco N., Santilli G., Francavilla S., Francavilla F. (2016). In vitro exposure of human spermatozoa to bisphenol A induces pro-oxidative/apoptotic mitochondrial dysfunction. Reprod. Toxicol..

[61] Chang M.X., Xiong F. (2020). Astaxanthin and its Effects in Inflammatory Responses and Inflammation-Associated Diseases: Recent Advances and Future Directions. Molecules.

[62] Ratliff B.B., Abdulmahdi W., Pawar R., Wolin M.S. (2016). Oxidant mechanisms in renal injury and disease. Antioxid. Redox Signal..

[63] Zeng Q., Yi H., Huang L., An Q., Wang H. (2019). Long-term arsenite exposure induces testicular toxicity by redox imbalance, G2/M cell arrest and apoptosis in mice. Toxicology.

[64] Zhao Z., Qu W., Wang K., Chen S., Zhang L., Wu D., Chen Z. (2019). Bisphenol A inhibits mucin 2 secretion in intestinal goblet cells through mitochondrial dysfunction and oxidative stress. Biomed. Pharmacother..

[65] Picard M., Taivassalo T., Gouspillou G., Hepple R.T. (2011). Mitochondria: Isolation, structure and function. J. Physiol..

[66] Blokhina O., Fagerstedt K.V. (2010). Oxidative metabolism, ROS and NO under oxygen deprivation. Plant Physiol. Biochem..

[67] Morgan E.T. (2001). Regulation of cytochrome p450 by inflammatory mediators: Why and how?. Drug Metab. Dispos..

[68] Guengerich F.P. (2001). Common and Uncommon Cytochrome P450 Reactions Related to Metabolism and Chemical Toxicity. Chem. Res. Toxicol..

